# Supplementary material for: Application of the Theoretical Framework of Acceptability to assess a telephone-facilitated health coaching intervention for the prevention and management of type 2 diabetes
Source: PLoS One. 2022 Oct 6;17(10):e0275576. doi: 10.1371/journal.pone.0275576 (PMC9536591; doi:10.1371/journal.pone.0275576)
Supplement: S2 Appendix — (PDF) [file pone.0275576.s002.pdf]

SMART2D Intervention
Point of Contact Structure

”Small changes can make a big difference!”

Working Version 2019-01-17

List of Points of Contact

1. Introduction meeting: Getting to know the program
   OBS! If baseline (HbA1c, BP, steps) not complete = F2F (+ hand over pedometer)/
   If baseline complete, leave pedometer at medborgarkontoret)
2. Physical activity: Increase physical activity in daily life/ reduce sedentary lifestyle
3. Diet: Regular meals and a balanced diet
4. Physical activity: Physical activity through the years
5. Diet: Fruit & vegetables
6. Physical activity: Increasing your physical activity levels
7. Diet: Sugar
8. Physical activity: Finding a physical activity that suits you
9. Concluding meeting: Summarize the experience of the hälsokompis program and discuss the way forward.

Session duration 30 mins

TOPIC: **Första kontakt**

MÅL MED MÖTET: Introduktion till möte 1/boka möte 1

Hej, jag heter ___ och jag ringer från karolinska institutet från diabetesprojektet som du är med i. Tidigare träffade du en av mina kollegor för en intervju som handlade mycket om mat och motion. Nu sätter vi i gång med näste steg i projektet.

Har du 15 minuter att prata med mig nu om vad projektet innebär?

[If NO: När kan jag ringa dig?]

Session duration 30 mins

ID #:
Diabetes/ Prediabetes/ Hög risk:
Typ av mobiltelefon:

FINDRISK:
HbA1c:
Steg:

Möte 1
Datum:
Kontaktperson:

ÄMNE: **Introduktionsmöte – Lär känna programmet**

MÅL MED MÖTET: Varför samarbeta med en hälsokompis för att göra livsstilsförändringar?

INTRODUKTION AV ÄMNET

Du är med i denna studie för att du har T2D/ har ökad risk för att få T2D. **Diskutera FINDRISKpoängen** (High risk)/ HbA1c (Prediabetes)/ T2D diagnosis. Små livsstilsförändringar kan göra stor skillnad - du mår bättre och du förbättrar din hälsa. Du som har hög risk för diabetes kan minska risken/ Du som har diabetes: kan kontrollera dina blodsockervärden och minska risken för diabeteskomplikationer (som stroke, hjärtinfarkt, försämrad syn, njursjukdom)

Vi vet att det kan vara svårt att göra livsstilsförändringar själv. Forskning visar att det kan vara till stor hjälp att göra saker med en annan person. Syftet med den här delen av projektet är att du ska samarbeta med en ”hälsokompis” för att göra livsstilsförändringar. Jag kommer att skicka dig ett paket med information om aktiviteter/övningar som ni kan göra tillsammans. Sedan kommer jag att ringa dig ungefär varannan vecka de närmsta tre månaderna för att guida dig igenom de olika aktiviteterna/övningarna. (9 telefonsamtal a 15-30 min)

- Hur känner du för att göra livsstilsförändringar? Som att förändra matvanor/öka fysisk aktivitet.
- Vad tycker du om idén att ha en ”hälsokompis”? Hur skulle en bra hälsokompis vara?
- Kan du tänka på någon som står dig nära som skulle kunna vara ett stöd för dig i att göra livsstilsförändringar? Om inte: fråga om de är intresserade av att vi sätter dem i kontakt med en annan deltagare i projektet? Ev info om gruppmöten
- För att skicka info behöver jag din adress………….. ………….

Forskning visar att sällskap kan vara till stor hjälp för att bli motiverad till att genomföra livsstilsförändringar som har positiva effekter på blodtryck och sockervärden

Aktivitet/Hemläxa

1) Välj en hälsokompis

2) Boka en träff med din hälsokompis. Gå igenom materialet tillsammans: Aktivitetsmanualen och broschyren “Information till dig som är en hälsokompis”. Diskutera hur ni vill samarbeta: ska ni träffas regelbundet? Hur ofta ska ni träffas? Vart ska ni träffas? Hur länge ska ni träffas? Vad tycker ni om de föreslagna aktiviteterna?

Utvärdering av mötet: Vad tyckte du om det här mötet? Är det något vi borde göra annorlunda nästa gång?

Datum för nästa möte:

Session duration 30 mins

ID #:

Diabetes/ Prediabetes/ Hög risk:

FINDRISK:

HbA1c:

Steg:

Möte 2

Datum:

Kontaktperson:

ÅTERKOPPLING FRÅN FÖREGÅENDE MÖTE (Vilka mål? Framsteg?)

- Har du valt en hälsokompis? Vem är det? Varför valde du den personen?
- Har du träffats med din hälsokompis för att diskutera hur ni ska jobba tillsammans? Vad har ni kommit fram till?
- Vad tycker du om aktiviteterna i programmet?

ÄMNE: **Öka fysisk aktivitet i vardagen/ minska stillasittande**

MÅL MED MÖTET: Vikten av fysisk aktivitet och hur den kan ökas i vardagen

INTRODUKTION AV ÄMNET

- Fysisk aktivitet har många fördelar! Du mår bättre, har mer energi, sover bättre och får lättare att koncentrera dig! Ditt immunsystem fungerar bättre och du lever längre! 30 minuters fysisk aktivitet (motsvarande en rask promenad) varje dag gör skillnad.
- Att sitta still större delen av dagen ökar risken för kronisk sjukdom
- Hur ser din fysiska aktivitet ut idag? Detta inkluderar familjeliv, hushållssysslor, trädgårdssysslor, arbetsuppgifter!
- Hur lång tid sitter du still varje dag?
- Vad skulle du kunna göra för att: öka fysisk aktivitet/minska stillasittande?

Exempel:

- ”Jag ska stå på ett ben när jag borstar tänderna”
- ”Jag ska alltid stå upp när jag pratar i telefon”
- ”Jag ska börja ta trapporna istället för hissen när jag ska till eller från min lägenhet”
- ”Jag ska gå av en hållplats tidigare när jag åker buss eller tunnelbana”

Kom ihåg att regelbundna, enkla aktiviteter som att promenera, gå i trappor eller städa kan bidra till minskning av blodtryck och blodsocker – Svenska Läkaresällskapet

Aktivitet/Hemläxa

- Diskutera förändringar du skulle vilja göra för att öka din dagliga aktivitetsnivå – bestäm 1-2 mål.
- Överväg att gå på en promenad med din hälsokompis. Vill du att det ska bli en regelbunden aktivitet?

Utvärdering av mötet:

Datum för nästa möte:

Session duration 30 mins

ID #:

Diabetes/ Prediabetes/ Hög risk:

FINDRISK:

HbA1c:

Steg:

Möte 3

Datum:

Kontaktperson:

ÅTERKOPPLING FRÅN FÖREGÅENDE MÖTE (Vilka mål? Framsteg?)

- Vilka mål satte du upp för att öka din dagliga aktivitetsnivå? Har du börjat? Hur går det? Om den fysiska aktiviteten inte har ökat: vad tror du skulle kunna hjälpa dig att komma igång?
- Har du gått på en promenad? Ensam eller med din hälsokompis? Kan du berätta om det? Kan du tänka dig att göra det regelbundet?
- Har du gjort några andra livsstilsförändringar? Berätta gärna.

ÄMNE: Hälsosamt ätande – **regelbundet, balanserat och nyttigt**

MÅL MED MÖTET: Vikten av att äta regelbundna, balanserade och nyttiga måltider

INTRODUKTION AV ÄMNET

- Hur ser dina matvanor ut idag?

- **Det är viktigt att äta regelbundna måltider (och minska småätande).**
- **Det är också viktigt att måltiderna är balanserade. Om du vill äta balanserade måltider kan du tänka på tallriksmodellen:** grönsaker/rotfrukter är största delen, andra delen är pasta/ris/potatis/bröd/ (välj helst fullkornsvarianter), och tredje delen: kött, ägg, fisk, baljväxter
- **Det är också viktigt att maten är nyttig, tex berätta om innehåll i manualen: mat man kan tänka på att öka/byta/begränsa.**
- **Ett lätt sätt att hitta nyttig mat i mataffären är att leta efter Nyckelhålsmärkta produkter.** Väljer du matvaror med Nyckelhålet får du i dig mat med mindre socker och salt, mer fullkorn och fibrer och nyttigare eller mindre fett.
- Vilka förändringar skulle du kunna göra för att få hälsosammare matvanor?
- Exempel: ”jag ska försöka äta frukost varje morgon” ”Jag ska byta från vitt bröd till fullkornsbröd”
- ”Jag ska byta från smör till vegetabilisk olja när jag steker mat” ”Jag ska inte ställa fram saltkaret på matbordet vid måltider”
- ”Jag ska tänka på tallriksmodellen när jag lägger upp/tillagar måltider”

Aktivitet/Hemläxa

- Gå till din vanliga mataffär med din hälsokompis. Diskutera vad du brukar köpa. Hur kan du göra dina matinköp hälsosammare?
- Bestäm 1-2 mål för att äta hälsosammare.

FÖRSLAG: leta efter/tänk ut ett hälsosamt recept som du och din hälsokompis kan laga tillsammans.

Utvärdering av mötet:

Datum för nästa möte:

Session duration 30 mins

ID #:

Diabetes/ Prediabetes/ Hög risk:

FINDRISK:

HbA1c:

Steg:

Möte 4

Datum:

Kontaktperson:

ÅTERKOPPLING FRÅN FÖREGÅENDE MÖTE (Vilka mål? Framsteg?)

- Har du besökt en matbutik med din hälsokompis? Hur var det? Har du lärt dig något om hur du kan äta mer hälsosamt?
- Vilka mål hade du satt upp för att äta mer hälsosamt? Har du börjat? Hur går det? Om dina matvanor inte har ändrats – vad tror du skulle kunna hjälpa dig att komma igång?
- Hur går det med dina mål att öka din dagliga aktivitetsnivå?
- Har du gjort några andra livsstilsförändringar? Berätta gärna.

ÄMNE: **Fysisk aktivitet genom åren**

MÅL MED MÖTET: Hur har din fysiska aktivitet ändrats genom åren?

INTRODUKTION AV ÄMNET

- **Vi har alla haft perioder I livet när vi har varit mer eller mindre fysiskt aktiva. Det är aldrig för sent att börja eller börja igen!**
- Tänk på hur nivån av din fysiska aktivitet har sett ut under olika faser av ditt liv (När du var barn? Tonåring? På olika jobb du haft? När du hade små barn? Nu?) **Detta inkluderar rutinmässiga dagliga aktiviteter (tex jobba, handling, städning, ta hand om barnen)!**
- Tänk på vilka typer av fysisk aktivitet som du har tyckt om? Finns det någonting du har testat som du skulle vilja börja med igen? Finns det något du har tänkt på att prova, men aldrig provat?
- **Facilitator note: Om deltagarna visar motstånd till att påbörja fysisk aktivitet – tänk på att motivera deltagarna istället föratt försöka övertala dem.**

Aktivitet/Hemläxa

- Rita en tidslinje över en viktiga faser i ditt liv (tex “när jag gick I skolan”; “när jag var tonåring”; när jag blev vuxen; ”när jag gifte mig/fick barn; osv.) Skriv ner all fysisk aktivitet du har utfört på tidslinjen.
- Diskutera med din hälsokompis: Vad har fysisk aktivitet inneburit i ert liv? När och varför har mängden eller formen av fysisk aktivitet ändrats? Kan ni tänka på en aktivitet som ni skulle vilja testa tillsammans för första gången eller har ni förslag på något ni kan ta upp igen?

Utvärdering av mötet:

Datum för nästa möte:

Session duration 30 mins

ID #:

Diabetes/ Prediabetes/ Hög risk:

FINDRISK:

HbA1c:

Steg:

Möte 5

Datum:

Kontaktperson:

ÅTERKOPPLING FRÅN FÖREGÅENDE MÖTE (Vilka mål? Framsteg?)

- Har du ritat en tidslinje och skrivit ner dina fysiska aktiviteter genom åren? Kan du berätta om dem?
- Har du diskuterat med din hälsokompis? Vad har fysisk aktivitet inneburit i ditt liv?
- Har ni diskuterat en aktivitet som ni skulle vilja testa tillsammans eller som ni vill prova att göra igen? Kan du berätta om det?
- Hur går det med dina mål att äta hälsosammare?
- Har du genomfört några andra livsstilsförändringar? Berätta gärna.

ÄMNE: Frukt och grönsaker

MÅL MED MÖTET: Vikten av att äta frukt och grönsaker varje dag

INTRODUKTION AV ÄMNET

Det rekommenderas att man ska äta minst två generösa nävar grönsaker och tre frukter varje dag (detta inkluderar inte potatis och juice).

Kom ihåg att all ökning är välgörande! Försök att variera frukten och grönsakerna du äter. Tips! Ett lätt sätt att variera är att ha grönsaker i olika färger på tallriken.

Hur ser ditt dagliga intag av frukt och grönsaker ut? Hur mycket? Hur ser variationen ut?

Hur kan du öka ditt dagliga intag av frukt och grönsaker? Göra det mer varierat?

Exempel:

- ”Jag ska ställa fram en skål frukt som alla i familjen ser”
- ”Jag ska börja servera en variation av (skurna/skivade/hackade) grönsaker tillsammans med kvällsmaten varje dag”
- ”Jag ska försöka börja använda en ny grönsak i min matlagning”

Även en liten ökning är bra för hälsan!
- Livsmedelsverket

Aktivitet/Hemläxa

- Diskutera med din hälsokompis om ditt nuvarande intag av frukt och grönsaker och vad du kan göra för att öka mängden och variationen. Bestäm ett eller två mål för hur du ska öka ditt intag av frukt/grönsaker.

Utvärdering av mötet:

Datum för nästa möte:

:

Session duration 30 mins

ID #:

Diabetes/ Prediabetes/ Hög risk:

FINDRISK:

HbA1c:

Steg:

Möte 6

Datum:

Kontaktperson:

ÅTERKOPPLING FRÅN FÖREGÅENDE MÖTE (Vilka mål? Framsteg?)

- Har du diskuterat med din hälsokompis vad du skulle kunna göra för att äta mer frukt och grönsaker? Kan du berätta om samtalet?
- Vilka mål satte du upp? Har du börjat? Hur går det? Om du inte har börjat äta mer frukt och grönsaker – vad tror du skulle kunna hjälpa dig att sätta igång?
- Hur går det med dina mål att öka din dagliga fysiska aktivitet?
- Har du gjort några andra livsstilsförändringar? Berätta gärna.

ÄMNE: **Öka mängden av din fysiska aktivitet**

MÅL MED MÖTET: Utmana dig själv att öka din dagliga fysiska aktivitet

INTRODUKTION AV ÄMNET

- I början av programmet fick du en stegräknare. Har du haft möjlighet att använda den? Varför inte? Vad är fördelarna? Nackdelarna? **TIPS! Om du har en Iphoone/Android så har du en hälsoapp som automatiskt räknar dina steg.**
- Vet du hur många steg du tar på en vanlig dag? Vet du hur många steg du går på dina mest aktiva dagar?
- **ALTERNATIV FÖR DOM SOM INTE VILL ANVÄNDA STEGRÄKNARE:** Bara 10 minuter av sammanhängande fysisk aktivitet har positiva hälsoeffekter! Vet du hur många gånger per dag du är fysiskt aktiv i 10 minuter i sträck?

Aktivitet/Hemläxa

1) Ha på dig din stegräknare under en vanlig dag och dokumentera hur många steg du tar. Utmana dig själv tillsammans med din hälsokompis – bestäm en dag och försök att öka antalet steg du tar. Skriv ner det!

ALTERNATIV: Skriv ner hur många gånger per dag du är aktiv i 10-minutersperioder. Bestäm en dag och försök att öka antalet sådana perioder.

Utvärdering av mötet:

Datum för nästa möte:

Session duration 30 mins

ID #:

Diabetes/ Prediabetes/ Hög risk:

FINDRISK:

HbA1c:

Steg:

Möte 7

Datum:

Kontaktperson:

ÅTERKOPPLING FRÅN FÖREGÅENDE MÖTE (Vilka mål? Framsteg?)

- Har du räknat hur många steg du tar en vanlig dag? Har du försökt att öka antalet steg du tar? Har du lärt dig något av den här övningen? Kan du tänka på några dagar som du går väldigt få steg? Vad hade du kunnat göra för att öka antalet steg? Om aktivitetsnivån inte har ökat - vad tror du skulle kunna hjälpa dig att komma igång?
- Hur går det med dina mål att äta hälsosammare?
- Har du gjort några andra livsstilsförändringar? Berätta gärna.

ÄMNE: Socker

MÅL MED MÖTET: Hur man kan minska dagligt intag av socker

INTRODUKTION AV ÄMNET

- Hur ser ditt dagliga intag av socker ut?

Kom ihåg att det finns dolt socker i maten vi äter, tex. ketchup, sylt, yoghurt, flingor och juice. Vet du sockermängden i maten du köper?

Om du har sötsug så är bästa sättet att motverka det genom att äta regelbundna måltider och ha en varierad kost/matvanor.

Hur kan du minska ditt dagliga intag av socker? Hur kan du ersätta det?

- Exempel:
- ”Jag ska börja äta frukost/3 måltider per dag”
- ”Jag ska ta mindre socker i mitt te/kaffe – en tesked istället för två”
- ”Jag ska sluta dricka sockrade drycker till maten”
- ”Jag ska köpa yoghurt/flingor med mindre socker”

Välj det söta med omsorg och njut av det - Livsmedelsverket

Aktivitet/Hemläxa

1) Diskutera med din hälsokompis om ditt nuvarande intag av socker och vad du skulle kunna göra för att minska/ersätta det. Överväg att kolla sockerinnehållet på några matvaror du brukar köpa.

2) Bestäm 1-2 mål för hur du ska minska ditt dagliga sockerintag.

Utvärdering av mötet:

Datum för nästa möte:

Session duration 30 mins

ID #:

Diabetes/ Prediabetes/ Hög risk:

FINDRISK:

HbA1c:

Steg:

Möte 8

Datum:

Kontaktperson:

ÅTERKOPPLING FRÅN FÖREGÅENDE MÖTE (Vilka mål? Framsteg?)

- Har du diskuterat din dagliga intag av socker med din hälsokompis? Vad har du lärt dig?
- Vilka mål satte du upp för att minska mängden socker du äter? Har du börjat? Hur går det? Om du inte har börjat äta mindre socker, vad tror du skulle hjälpa dig att sätta igång?
- Hur går det med dina mål att äta hälsosammare?
- Hur går det med dina mål att öka din dagliga fysiska aktivitet?
- Har du gjort några andra livsstilsförändringar? Berätta gärna.

ÄMNE: **Hitta en fysisk aktivitet som passar dig**

MÅL MED MÖTET: Att öka fysisk aktivitet i vardagen genom att göra det roligt

INTRODUKTION AV ÄMNET

- **Det är mycket lättare att vara fysiskt aktiv om du hittar en träningsform som passar dig!**
- Det finns många sätt att vara aktiv. Vad tycker du är kul? Vilka intressanta/roliga möjligheter för fysisk träning finns tillgängliga där du bor? Kan du beskriva ditt område utifrån de möjligheter som finns för fysisk aktivitet?

Exempel:

- Promenadvägar?
- Badhus?
- Gym?
- Bollsport?
- Dans?

Aktivitet/Hemläxa

1) Diskutera med din hälsokompis om vilka fysiska aktiviteter som ni kan testa tillsammans.

2) Besök/testa en av dessa aktiviteter tillsammans. Lägg märke till de positiva aspekterna – hur är miljön/atmosfären? Hur känner du dig innan och efter, både fysiskt och mentalt?

Utvärdering av mötet:

Datum för nästa möte:

Session duration 30 mins

ID #:

Diabetes/ Prediabetes/ Hög risk:

FINDRISK:

HbA1c:

Steg:

Möte 9 (Sista mötet)

Datum:

Kontaktperson:

ÅTERKOPPLING FRÅN FÖREGÅENDE MÖTE (Vilka mål? Framsteg?)

- Har du provat en ny aktivitet med din hälsokompis? Vad gjorde ni? Hur gick det? Kommer ni att fortsätta?
- Har du gjort några andra livsstilsförändringar? Berätta gärna.

ÄMNE: Hälsosam livsstil framöver

MÅL MED MÖTET: Hur har det gått med att ändra till en hälsosammare livsstil tillsammans med en hälsokompis? Hur ska ni fortsätta/stärka relationen i framtiden?

HUR LÅNGT HAR DU KOMMIT OCH HUR SKA DU FORTSÄTTA

Se över programmet och hjälp deltagaren att hitta/notera fördelarna med att göra livsstilsförändringarna och uppmärksamma sin bedrift/insats:

- Hur går det med målen du har satt upp för dig själv för att få hälsosammare matvanor? Hur känns det?
- Hur går det med målen du satt upp för att öka din dagliga fysiska aktivitet? Hur känns det?
- Vad tror du skulle hjälpa dig att upprätthålla förändringarna som du har gjort?
- Hur har det varit att ha en hälsokompis? Har det hjälpt dig att göra din livsstil hälsosammare? Berätta
- Planerar du att fortsätta att samarbeta med din hälsokompis? Hur skulle du vilja att er relation utvecklades i framtiden? Vad kan du/ni göra för att förstärka samarbetet?
- Vilket annat stöd tror du skulle vara hjälpsamt för dig för att uppnå dina hälsosamma livsstilmål?

Aktivitet/Hemläxa

- Reflektera över tiden som du har samarbetat med en hälsokompis för att förändra dina livsstilsval. Vad har du uppnått/fått ut? Hur får det dig att känna? Hur får det dig att se på framtiden?

Tack för ditt deltagande!

Timeline for Contact Points:

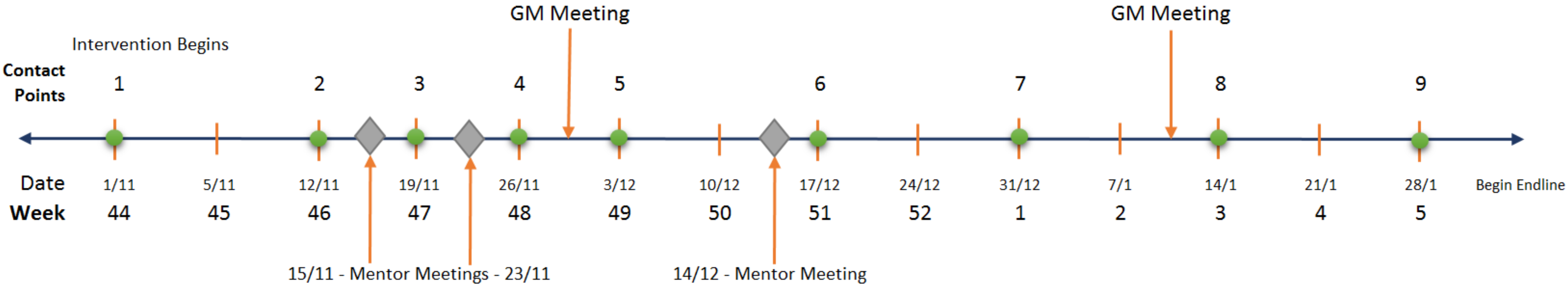

- Green dot indicates a scheduled session (1-9)
- Facilitators will record one session per contact point (not the first session)
- Grey diamond indicates the three mentor sessions that will take place with the facilitators in order to discuss the process of facilitation and identify areas in the FCG that needed to be amended/modified
- A Group meeting (GM) will be scheduled at 2 sites (Hässelby & Tenesta) between contact point 4 & 5 and 7 & 8.

UPDATED VERSION

Session duration 30 mins

ID #:
Diabetes/ Prediabetes/ Hög risk:

FINDRISK:
HbA1c:
Steg:

Möte 9 (Sista mötet)
Datum:
Kontaktperson:

ÅTERKOPPLING FRÅN FÖREGÅENDE MÖTE (Vilka mål? Framsteg?)

- Har du diskuterat/ provat en ny aktivitet med din hälsokompis? Vad gjorde ni? Hur gick det? Kommer ni att fortsätta?

ÄMNE: **Avslutande möte: Sammanfatta upplevelsen av hälsokompis-programmet och diskutera vägen framåt**

MÅL MED MÖTET: Hur har det gått med att ändra till en hälsosammare livsstil tillsammans med en hälsokompis? Hur ska du fortsätta?

Se över programmet och hjälp deltagaren att hitta/notera fördelarna med att göra livsstilsförändringarna med sin hälsokompis OCH uppmärksamma dens bedrift/insats:

- Hur går det med målen du satt upp för att öka din dagliga fysiska aktivitet? Hur känns det?
- Hur går det med målen du har satt upp för dig själv för att få hälsosammare matvanor? Hur känns det?
- Om du tanker på de olika mötena i programmet, fanns det några ämnen/aktiviteter som du ansåg vara mer givande än andra? Kan du beskriva.
- Fanns det några ämnen/aktiviteter du upplevde som mindre givande. Kan du beskriva.
- **OM inte HK:** Du hittade ingen hälsokompis och ville inte paras ihop med en annan deltagare/ Vi lyckades inte para ihop dig. Tror du att det hade hjälp dig nå dina mål om du hade haft en hälsokompis? Varför/ Varför inte?
- **OM HK:** Hur har det varit att ha en hälsokompis? Har det hjälpt dig att göra din livsstil hälsosammare? Berätta
- **OM HK:** Planerar du att fortsätta att samarbeta med din hälsokompis? Hur skulle du vilja att er relation utvecklades i framtiden? Vad kan du/ni göra för att förstärka samarbetet?
- Vilket annat stöd tror du skulle vara hjälpsamt för dig för att uppnå dina hälsosamma livsstilmål?

Nästa sted

- **Avtala tid**: **Om behörig!** En (ca 10 min) telefonintervju med en kollega om deltagarens upplevelse av telefonmötena.
- **Informera**: **Om behörig!** Under de kommande veckorna kommer vi att ringa dig för att avtala tid för den sista projektintervjun (samma som den i början på projektet). Som tack vill vi ge dig två biobiljetter.
- **Informera**: Vi håller på att planera en andra Inspirationskväll med möjlighet att lyssna på experter och träffa representanter från VC, medborgarkontoret och projektteamet så väl som andra deltagare. Vi kommer snart att skicka mer information.
- **Fråga**: **Om behörig!** Skulle du vara intresserad av at delta om vi fortsätter med detta projekt i någon form? (uppföljning/ gruppmöten)

[illegible]

Session duration 10 mins

ID #:

Diabetes/ Prediabetes/ Hög risk:

FINDRISK:

HbA1c:

Steg:

Uppföljningssamtal

Datum:

Kontaktperson:

Samtal 1: En månad efter att interventionen avslutats

Samtal 2:

ÄMNE: Uppföljningssamtal

MÅL MED MÖTET: säkerställa kontinuitet, hålla deltagaren engagerad och förtydliga eventuella frågor.

- Hur går det med din fysiska aktivitet efter programmet tagit slut?
- Hur går det med dina matvanor?
- Har du haft några svårigheter med matvanor, fysisk aktivitet eller hälsa?
- Har du några frågor som jag kan hjälpa till att förtydliga?
