# Supplementary material for: Application of the Theoretical Framework of Acceptability to assess a telephone-facilitated health coaching intervention for the prevention and management of type 2 diabetes
Source: PLoS One. 2022 Oct 6;17(10):e0275576. doi: 10.1371/journal.pone.0275576 (PMC9536591; doi:10.1371/journal.pone.0275576)
Supplement: S3 Appendix — (PDF) [file pone.0275576.s003.pdf]

SMART2D Intervention
Point of Contact Structure

”Small changes can make a big difference!”

Working Version 2019-01-17

List of Points of Contact

1. Introduction meeting: Getting to know the program
   OBS! If baseline (HbA1c, BP, steps) not complete = F2F
2. Physical activity: Increase physical activity in daily life/ reduce sedentary lifestyle
3. Diet: Healthy eating – Regular meals, balanced meals, healthy choices
4. Physical activity: Physical activity through the years
5. Diet: Fruit & vegetables
6. Physical activity: Increasing your physical activity levels
7. Diet: Sugar
8. Physical activity: Finding a physical activity that suits you
9. Concluding meeting: Summarize the experience of the hälsokompis program and discuss the way forward.

Session duration 30 mins

TOPIC: **Initial contact**

GOAL OF MEETING: Introduction to Session 1/ Call to book session 1.

Hi my name is ____________and I am calling from Karolinska Institutet. I work with the SMART2D diabetes project in which you are participating. You have previously met with myself or one of my colleagues for an interview focused on diet and physical activity. We are now about the start the next step of the project.

Do you have 15 minutes to speak to me about what the project entails?

[if NO: When can I ring you?]

Session duration 30 mins

ID #:
Diabetes/ Prediabetes/ High risk:
Type of mobile phone

FINDRISK:
HbA1c:
BP:
Steps:

Session 1
Date:
Facilitator:

TOPIC: **Introduction meeting - Getting to know the program**

GOAL OF MEETING: Why work with a ”hälsokompis” to make lifestyle changes?

TOPIC INTRODUCTION

You have been invited to participate in this study because you have T2D/ prediabetes/ elevated risk for developing diabetes: Discuss FINDRISK score (High risk)/ HbA1c (Prediabetes)/ T2D diagnosis. Small lifestyle changes can make a big difference – you feel better and you improve your health. If you have elevated risk of developing T2D you can reduce this risk. If you have diabetes you can better control your blood sugar levels and reduce your risk of diabetes complications (such as stroke, heart attack, reduced eyesight, kidney disease).

We know that it can be difficult to make lifestyle changes on your own. Research shows that it can be of great benefit to work together with somebody else. The aim of this part of the project is for you to work with a so called "hälsokompis" or "health buddy" to make lifestyle changes. I will send you a packet including a manual outlining activities/ exercises that you can do together. I will then ring you every other week for the next few months to guide you through these activities/ exercises (9 telephone conversations x 15-20 minutes)

- How do you feel about making changes to your diet? Increasing your physical activity?
- What do you think about the idea of having a "health buddy"? What would make a good hälsokompis?
- Can you think of somebody close to you who can support you in making lifestyle changes?

If NO: Ask if they would be interested in being paired up with another participant?

Inform about group meetings

To send you the information pack, I will need your address__________________

Forskning visar att sällskap kan vara till stor hjälp för att bli motiverad till att genomföra livsstilsförändringar som har positiva effekter på blodtryck och sockervärden

Activity/ Homework

1) Appoint a hälsokompis

2) Arrange a meeting with your hälsokompis. Go through the material (Activity Manual & "Information till dig som är en hälsokompis"). Discuss how you would like to work together: Will you meet regularly? How often will you meet? Where will you meet? For how long will you meet? What do you think of the activities suggested?

Do you have any questions about the activities?

Session evaluation: What did you think about this session? Is there anything you would like to do differently next time?

Date of next meeting:

Session duration 30 mins

ID #:
Diabetes/ Prediabetes/ High risk:
Type of mobile phone

FINDRISK:
HbA1c:
BP:
Steps:

Session 2
Date of contact:
Facilitator:

FEEDBACK FROM LAST SESSION (Which goals? Progress?)

- Have you received the information package!!!!!
- Have you appointed a hälsokompis? Who is it? Why did you choose this person?
- Have you met up with your hälsokompis to discuss how you will work together? What have you decided?
- What do you think of the activities in the program?

TOPIC: **Increase physical activity in daily life/ reduce sedentary lifestyle**

GOAL OF MEETING: The importance of physical activity and how this can be increased in daily life

TOPIC INTRODUCTION

Physical activity has many benefits! You feel better, have more energy, sleep better and find it easier to concentrate! Your immune system works better and you live longer! 30 minutes of activity (equivalent to a brisk walk) a day makes a difference.

Sitting still for a large part of the day increases the risk of chronic disease!

- What does your daily physical activity look like today? **Includes family activities/ household chores/ garden work/ work tasks!**
- For how long do you sit still every day?

What would you be able to to increase physical activity/ reduce sitting still?

Examples:

- ”I will stand on one leg when I brush my teeth”
- “I will start to always stand up when I am talking on the phone”
- “I will start to take the stairs instead of the lift in my appartment building”
- ”I will get off the bus/ train one stop earlier”

Kom ihåg att regelbundna, enkla aktiviteter som att promenera, gå i trappor eller städa kan bidra till minskning av blodtryck och blodsocker – Svenska Läkaresällskapet

Activity/ Homework

1) Discuss changes you would like to make to increase your daily activity level – Agree on 1-2 goals.

2) Consider going on a walk with your hälsokompis. Do you want to make this a regular activity?

Session evaluation:

Date of next meeting:

Session duration 30 mins

ID #:
Diabetes/ Prediabetes/ High risk:
Type of mobile phone

FINDRISK:
HbA1c:
BP:
Steps:

Session: 3
Date of contact:
Facilitator:

FEEDBACK FROM LAST SESSION (Which goals? Progress?)

- What goals did you set to increase your daily activity level? Have you started? How is it going? If activity has not increased: What do you think would help you get started?
- Did you go on a walk? Alone or with your hälsokompis? Can you tell me about it? Would you consider doing regularly?
- Have you made any other lifestyle changes? Please describe.

TOPIC: **Healthy eating – Regular meals, balanced meals, healthy choices**

GOAL OF MEETING: The importance of eating regular and balanced meals and making healthy choices

TOPIC INTRODUCTION

- What does your diet look like today?

It is important to eat regular meals (and limit snacking).

It is also important that your meals are <u>balanced</u>. If you want to eat balanced meals, think about "tallriksmodellen" or the "plate model": Vegetables represent the largest proportion, followed by carbohydrates (pasta/rice/ potatoes/ bread (try to eat wholegrain alternatives!) and the third consists of meat, eggs, fish and legumes (lentils, beans and peas)

It is also important to make healthy choices (See your manual for examples of foods that you should eat more of/ switch to healthier alternatives/ limit. An easy way to find healthy options at the grocery store is to look for "nyckelhålet" or "the keyhole". These products are low in sugar and salt, low in refined products (with more whole meal and fiber) and with healthier or less fat!

What changes could you potentially make to have a healthier diet?

Examples: "I will try to eat breakfast every morning", "I will switch from white bread to wholemeal bread";

"I will switch from butter to vegetable oil when I fry food"; I will avoid placing the salt cellar on the table on the table during meals",

" I will think about "the plate model" when I make/serve food"

Activity/ Homework

1) **Go to your local supermarket with your hälsokompis. Discuss what you usually buy. How can you make your food purchases healthier?**
2) **Set 1-2 goals for how you can make your food purchases healthier.**

SUGGESTION: Look for/ figure out a healthy recipe that you and your hälsokompis can prepare together.

Session evaluation:

Date of next meeting:

Session duration 30 mins

ID #:

Diabetes/ Prediabetes/ High risk:

Type of mobile phone

FINDRISK:

HbA1c:

BP:

Steps:

Session 4

Date of contact:

Facilitator:

FEEDBACK FROM LAST SESSION (Which goals? Progress?)

- Have you visited a local supermarket with your hälsokompis? How was the experience? Have you learned anything about how to have a more healthy and balanced diet?
- What goals did you set to have a healthier diet? Have you started? How is it going? If food habits have not changed: What do you think would help you get started?
- How is it going with your goals to increase your daily activity levels?
- Have you made any other lifestyle changes? Please describe

TOPIC: Physical activity through the years

GOAL OF MEETING: How has your physical activity levels changed over the years?

TOPIC INTRODUCTION

We have all had periods of time in our lives when we have been more or less active. It is never to late to start/start again!

Think about your physical activity levels during different phases of your life (when you were a child? A teenager? At the different jobs you have had? When you had small children? Now?). **This includes routine daily activities (e.g. working, shopping, cleaning, caring for children)!**

Think about what types of physical activity you enjoyed? Is there anything that you have tried that you would like to start with again? Is there anything you have thought of trying, but never did?

Activity/ Homework

1) **Draw a timeline with important phases of your life (e.g. ”when I went to school”; ”when I was a teenager”; ”when I became an adult”; ”when I got married/ had children”; etc.) Note all physical activity you have performed on the timeline**
2) **Discuss with your hälsokompis: What has physical activity entailed in your life? When and why has change occurred in the amount of type of activity? Can you think of an activity that you would like to try together/ try to do again?**

Session evaluation:

Date of next meeting:

Session duration 30 mins

ID #:

Diabetes/ Prediabetes/ High risk:

Type of mobile phone

FINDRISK:

HbA1c:

BP:

Steps:

Session 5

Date of contact:

Facilitator:

FEEDBACK FROM LAST SESSION (Which goals? Progress?)

- Have you drawn a time line and noted your physical activity throughout the years? Can you tell me about these?
- Have you discussed with your hälsokompis? What has physical activity entailed in your life?
- Have you discussed an activity that you would like to try to do together/ try to do again? Can you tell me about it?
- How is it going with your goals to have a more healthy / balanced diet?
- Have you made any other lifestyle changes? Please describe

TOPIC: Fruit and vegetables

GOAL OF MEETING: The importance of eating fruit & vegetables every day

TOPIC INTRODUCTION

It is recommended that we eat a least 2 generous handfulls of vegetables and 3 pieces of fruit every day (Note that this does not include potatoes/ juice).

Remember that any increase is beneficial! Try to vary the fruit & vegetables you eat. Increasing the number of colours on your plate is said to contribute to a more healthy meal!

What does your daily intake of fruit and vegetable intake look like? What amount? What variety?

How could you increase your daily intake of fruit and vegetables? Make it more varied?

Exempel:

- “I will put out a fruit bowl for everyone in the family to see”
- ”I will start serving a variety of chopped vegetables with the evening meal each day”
- ”I will try to use a new vegetable in my cooking”

Även en liten ökning är bra för hälsan!
- Livsmedelsverket

Activity/ Homework

1) **Discuss with your hälsokompis about your current intake of fruit and vegetables and what you might be able to do to increase it. Set a goal or two for increasing your daily fruit/ vegetable intake.**

Session evaluation:

Date of next meeting:

Session duration 30 mins

ID #:

Diabetes/ Prediabetes/ High risk:

Type of mobile phone

FINDRISK:

HbA1c:

BP:

Steps:

Session 6

Date of contact:

Facilitator:

FEEDBACK FROM LAST SESSION (Which goals? Progress?)

- Did you discuss with your hälsokompis what you might be able to do to increase your intake of fruit and vegetables? Can you tell me about the discussion?
- What goals did you set? Have you started? How is it going? If fruit/ vegetable intake has not increased: What do you think would help you get started?
- How is it going with your goals to increase your daily activity levels?
- Have you made any other lifestyle changes? Please describe

TOPIC: **Increasing your physical activity levels**

GOAL OF MEETING: Challenge yourself to increase daily physical activity

TOPIC INTRODUCTION

- You received a pedometer at the start of the program. Have you had a chance to use it? Why not? What are the benefits? Drawbacks? **TIP! If you have an Iphone/android you have a health app that automatically counts steps for you**

- Do you know how many steps you take on an average day? Do you know how many steps you take on your most active days?

ALTERNATIVE for those not wanting to use pedometer: Just 10 minutes of continuous activity has cardio-metabolic benefits. Do you know how many times you are physically active for a period of 10 minutes during the day?

Activity/ Homework

1) **Wear your pedometer during an average day and document how many steps you take. Set yourself a challenge together with your hälsokompis – Choose a day and try to increase the number of steps you take. Record!**

ALTERNATIVE: Note how many times a day you are active for 10 minute periods. Choose a day and try to increase the number of these active periods.

Session evaluation:

Date of next meeting:

Session duration 30 mins

ID #:
Diabetes/ Prediabetes/ High risk:
Type of mobile phone

FINDRISK:
HbA1c:
BP:
Steps:

Session 7
Date of contact:
Facilitator:

FEEDBACK FROM LAST SESSION (Which goals? Progress?)

- Have you counted the steps you take on an average day? Have you tried to increase the number of steps you take? Have you learned anything from this exercise? Can you think of any days when you take very few steps? What could you have done to increase the number of steps? If activity level has not increased: What do you think would help you get started?
- How is it going with your goals to have a healthy / balanced diet?
- Have you made any other lifestyle changes? Please describe

TOPIC: Sugar

GOAL OF MEETING: How sugar consumption can be decreased in daily life

TOPIC INTRODUCTION

- What does your daily intake of sugar look like?

Remember that there is hidden sugar in the food we eat e.g. Ketchup, jam, yoghurt cereal and juice! Do you know the sugar content of your food purchases?

If you have sugar cravings, these are best tackled by eating regular meals and having a varied diet!

How could you decrease your daily intake of sugar? How could you replace it?

Exempel:

- ”I will start to eat breakfast/ 3 meals a day”
- “I will start to take less sugar in my tea/ coffee– 1 teaspoon instead of two”
- ”I will stop drinking sugary drinks with meals”
- ”I will by a yoghurt/ cereal with less sugar”

Välj det söta med omsorg och njut av det - Livsmedelsverket

Activity/ Homework

1) **Discuss with your hälsokompis about your current intake of sugar and what you might be able to do to decrease it/ replace it. Consider checking the sugar content on some of the foods you normally eat.**
2) **Set 1-2 goals for decreasing your daily sugar intake.**

Session evaluation:

Date of next meeting:

Session duration 30 mins

ID #:

Diabetes/ Prediabetes/ High risk:

Type of mobile phone

FINDRISK:

HbA1c:

BP:

Steps:

Session 8

Date of contact:

Facilitator:

FEEDBACK FROM LAST SESSION (Which goals? Progress?)

- Have you discussed with your hälsokompis about your current intake of sugar. What have you learnt?
- What goals did you set to decrease the amount of sugar you eat? Have you started? How is it going? If sugar intake has not decreased: What do you think would help you get started?
- How is it going with your goals to have a healthy / balanced diet?
- How is it going with your goals to increase your daily activity levels?
- Have you made any other lifestyle changes? Please describe

TOPIC: Finding a physical activity that suits you

GOAL OF MEETING: The importance of physical activity and how this can be increased in daily life by making it fun

TOPIC INTRODUCTION

It is much easier to be physically active if you find a form of exercise that suits you!

There are many was of being active. What do you think is fun? What interesting/ fun opportunities for physical exercise are available in your area? Can you describe your area in terms of the opportunities for physical activity?

Examples:

- Walking tracks?
- Swimming pool?
- Gym?
- Ball games?
- Dance?

Activity/ Homework

1) Discuss with your hälsokompis about physical activities that you could try together.

2) Visit/ try one of these activities together. Pay attention to the positive aspects: How is the atmosphere? How you feel during and after, both physically and mentally?

Session evaluation:

Date of next meeting:

Session duration 30 mins

ID #:

Diabetes/ Prediabetes/ High risk:

Type of mobile phone

FINDRISK:

HbA1c:

BP:

Steps:

Session 9 (LAST SESSION):

Date of contact:

Facilitator:

FEEDBACK FROM LAST SESSION (Which goals? Progress?)

- Have you tried a new activity with your hälsokompis? What did you do? How did it go? Will you continue?
- Have you made any other lifestyle changes? Please describe

TOPIC: Healthy lifestyle moving forward

GOAL OF MEETING: How has it been to adopt a healthier lifestyle with your hälsokompis? How will you continue/ strengthen your relationship in the future?

HOW FAR HAVE YOU COME AND HOW WILL YOU CONTINUE?

Summarise the program and help the participant to find/note the advantages of making lifestyle changes ALSO acknowledge the progress they have made!

- How is it going with the goals that you have set for yourself with regard to having a healthier diet? How does it feel?
- How is it going with the goals you have set for yourself with regard to increasing your daily physical activity? How does it feel?
- What do you think would help you maintain your progress?
- How has it been to have a hälsokompis? Has it helped you make your lifestyle healthier? Describe.
- Do you expect to continue working with your hälsokompis? How would you like to see your relationship develop in the future?
- What could you do to promote a better collaboration with your hälsokomis?
- What other support do you think would be helpful to achieve your healthy lifestyle goals?

Activity/ Homework

Reflect on the time during which you have worked with a hälsokompis in order to modify your lifestyle choices. What have you gained? How does this make you feel? How does it make you look at the future?

Thank you for your participation!

Timeline for Contact Points:

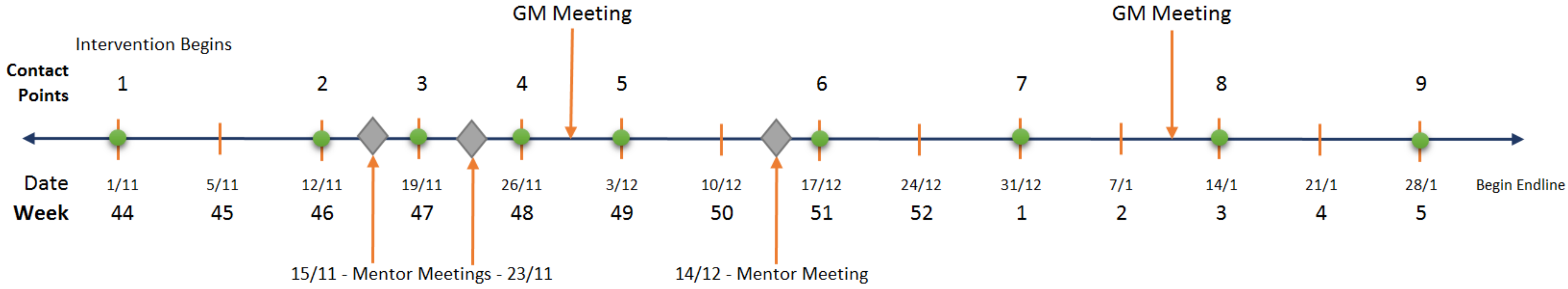

- Green dot indicates a scheduled session (1-9)
- Facilitators will record one session per contact point (not the first session)
- Grey diamond indicates the three mentor sessions that will take place with the facilitators in order to discuss the process of facilitation and identify areas in the FCG that needed to be amended/modified
- A Group meeting (GM) will be scheduled at 2 sites (Hässelby & Tenesta) between contact point 4 & 5 and 7 & 8.

Session duration 30 mins

UPDATED VERSION

ID #:
Diabetes/ Prediabetes/ High risk:
Type of mobile phone

FINDRISK:
HbA1c:
BP:
Steps:

Session 9 (LAST SESSION):
Date of contact:
Facilitator:

FEEDBACK FROM LAST SESSION (Which goals? Progress?)

- Have you discussed trying/ tried a new activity with your hälsokompis? What did you do? How did it go? Will you continue?

TOPIC: Summarize the experience of the hälsokompis program and discuss the way forward.

GOAL OF MEETING: How has it been to adopt a healthier lifestyle with your hälsokompis? How will you continue?

Summarise the program and help the participant to find/note the advantages of making lifestyle changes ALSO acknowledge the progress they have made!

- How is it going with the goals you have set for yourself with regard to increasing your daily physical activity? How does it feel?
- How is it going with the goals that you have set for yourself with regard to having a healthier diet? How does it feel?
- If you think about the different meetings in the program, were there any subjects or activities that your considered more beneficial than others? Please describe.
- Were there any subjects or activities that you found less beneficial. Please describe
- **If NO HC:** You did not find a hälsokompis and did not want to be paired with another participant/ we were unable to pair you. Do you think that it would have helped you to reach your goals if you had had a hälsokompis? Why/ Why not?
- **If HC:** How has it been to have a hälsokompis? Has it helped you make your lifestyle healthier? Describe.
- **If HC:** Do you expect to continue working with your hälsokompis? How would you like to see your relationship develop in the future? What could you do to promote a better collaboration with your hälsokomis?
- What other support do you think would be helpful to achieve your healthy lifestyle goals in the future?

Next steps

- **Schedule**: **If eligible!** A (Ca. 10 min) call with colleague to ask a questions about participant's experience of the telephone sessions.
- **Inform**: **If eligible!** In the coming weeks, we will call to schedule the final project interview (same as the one you did at the start of the project). As a thank you we would like to give you two cinema tickets.
- **Inform**: We are planning a second Inspirationskväll with the opportunity to listen to experts and meet representatives from VC, medborgarkontoret and the project team as well as other participants. We will send information shortly.
- **Enquire**: **If eligible!** Would you be interested in participing if we continue with this project in some form? (Follow-up, group meetings)

Session duration 10 mins

ID #:
Diabetes/ Prediabetes/ High risk:
Type of mobile phone:

FIFINDRISK:
HbA1c:
BP:
Steps:

Follow-up call 1 and 2
Date of contact:
Facilitator:

Call 1: one month after intervention has finalized
Call 2:

TOPIC: **Follow-up call**
GOAL OF MEETING: to ensure continuity, keep the participant engaged and clarify potential questions.

- How is your physical activity since we finished the project?
- How is your diet?
- Have you had any issues in relation to diet, physical activity or health?
- Do you have any questions that I can help clarifying?
