# Supplementary material for: Application of the Theoretical Framework of Acceptability to assess a telephone-facilitated health coaching intervention for the prevention and management of type 2 diabetes
Source: PLoS One. 2022 Oct 6;17(10):e0275576. doi: 10.1371/journal.pone.0275576 (PMC9536591; doi:10.1371/journal.pone.0275576)
Supplement: S4 Appendix — (DOCX) [file pone.0275576.s004.docx]

**S4 Appendix**: Reported responses (%) to the acceptability questionnaire

| **Questions** | **Completely**  **Disagree (%)** | **Disagree**  **(%)** | **Neutral**  **(%)** | **Agree**  **(%)** | **Completely**  **Agree (%)** |
| --- | --- | --- | --- | --- | --- |
| This program has helped me to eat healthier | 4 | 2 | 2 | 26 | 66 |
| The possibility for support from others besides healthcare providers is important for me | 6 | 2 | - | 14 | 78 |
| I have enjoyed the discussions with the facilitator | 2 | - | - | 8 | 90 |
| This program has helped me to increase physical activity in my daily life | 2 | 8 | 4 | 28 | 58 |
| It has been easy to understand how this program can help me | 2 | 4 | - | - | 94 |
| I have appreciated the activities suggested in the sessions | 6 | - | 2 | 24 | 68 |
| I feel my health is better now compared to when I started the program | 4 | 16 | 12 | 18 | 50 |
| The activities in this program have fitted well with how I want to live my life | - | - | 6 | 20 | 74 |
| I am glad that I was asked to participate in this program | - | 2 | - | 18 | 80 |
| I feel that I have achieved the goals set together with my facilitator or my care companion | - | 6 | 8 | 42 | 44 |
| I am confident I can continue the new habits discussed with my FT in my daily life | - | 6 | 2 | 18 | 74 |
|  |  |  |  |  |  |
| I feel that I have received enough information about the program | 2 | - | 2 | 10 | 86 |
| The length of the sessions was not too long | 2 | 2 | 2 | 6 | 88 |
| I feel that I have received enough information about SMART2D | 6 | 2 | - | 20 | 72 |
| It has been easy and effortless to have the sessions on phone | - | 10 | - | 10 | 80 |
|  |  |  |  |  |  |
| I have spent less time with my family/friends due to participation in the program | 12 | 14 | - | 4 | 70 |
| I have changed my schedule to be able to participate in the coaching sessions | 20 | 20 | - | 4 | 56 |
