# Supplementary material for: Application of the Theoretical Framework of Acceptability to assess a telephone-facilitated health coaching intervention for the prevention and management of type 2 diabetes
Source: PLoS One. 2022 Oct 6;17(10):e0275576. doi: 10.1371/journal.pone.0275576 (PMC9536591; doi:10.1371/journal.pone.0275576)
Supplement: Attachment — Submitted filename: Response to Reviewers.docx [file pone.0275576.s006.docx]

| **Revision** | **Response** |
| --- | --- |
| 1.) Please ensure that your manuscript meets PLOS ONE's style requirements, including those for file naming. The PLOS ONE style templates can be found at  <https://journals.plos.org/plosone/s/file?id=wjVg/PLOSOne_formatting_sample_main_body.pdf> and  <https://journals.plos.org/plosone/s/file?id=ba62/PLOSOne_formatting_sample_title_authors_affiliations.pdf> | Revisions done according to the guidelines. |
| 2.) Please include additional information regarding the coaching material used in the study and ensure that you have provided sufficient details that others could replicate the analyses. If you developed material for the telephone-facilitated health coaching session as part of this study and it is not under a copyright more restrictive than CC-BY, please include a copy, in both the original language and English, as Supporting Information, or include a citation if it has been published previously. | Supplementary material added:  - S2_Appendix SMART2D intervention guide_Points of Contact and SOPs SWEDISH.  - S3_Appendix SMART2D Intervention guide _Points of Contact and SOPs ENGLISH.  - Captions below the heading Supporting information on page 21, lines 583-587. |
| 3.) We note that you have indicated that data from this study are available upon request. PLOS only allows data to be available upon request if there are legal or ethical restrictions on sharing data publicly. For information on unacceptable data access restrictions, please see <http://journals.plos.org/plosone/s/data-availability#loc-unacceptable-data-access-restrictions>.  In your revised cover letter, please address the following prompts:  a) If there are ethical or legal restrictions on sharing a de-identified data set, please explain them in detail (e.g., data contain potentially identifying or sensitive patient information) and who has imposed them (e.g., an ethics committee). Please also provide contact information for a data access committee, ethics committee, or other institutional body to which data requests may be sent.  b) If there are no restrictions, please upload the minimal anonymized data set necessary to replicate your study findings as either Supporting Information files or to a stable, public repository and provide us with the relevant URLs, DOIs, or accession numbers. Please see <http://www.bmj.com/content/340/bmj.c181.long> for guidelines on how to de-identify and prepare clinical data for publication. For a list of acceptable repositories, please see <http://journals.plos.org/plosone/s/data-availability#loc-recommended-repositories>.  We will update your Data Availability statement on your behalf to reflect the information you provide. | The minimal anonymized data set necessary to replicate our study findings has been added as a Supporting Information file titled ‘S5 Appendix SMART2D Acceptability raw data’. |
| 4.) Thank you for stating the following in the Financial Disclosure section:  'This study is part of the SMART2D project supported by the EU Horizon 2020 Health Coordination Activities (Grant Agreement No 643692), MD, under call HCO-05-2014 (Global Alliance for Chronic Diseases: Prevention and treatment of type 2 diabetes) URL: <https://ec.europa.eu/programmes/horizon2020/en/h2020-sections-projects>; and partially funded by Region Stockholm’s Public Healthcare Services Administration (Hälso- och sjukvårdsförvaltningen), URL: <https://www.sll.se/om-regionstockholm/Organisation/forvaltningar/halso-och-sjukvardsforvaltningen/>. The funders had no role in study design, data collection and analysis, decision to publish, or preparation of the manuscript.'  We note that one or more of the authors are employed by a commercial company: Collaborative Care Systems Finland  a. Please provide an amended Funding Statement declaring this commercial affiliation, as well as a statement regarding the Role of Funders in your study. If the funding organization did not play a role in the study design, data collection and analysis, decision to publish, or preparation of the manuscript and only provided financial support in the form of authors' salaries and/or research materials, please review your statements relating to the author contributions, and ensure you have specifically and accurately indicated the role(s) that these authors had in your study. You can update author roles in the Author Contributions section of the online submission form.  Please also include the following statement within your amended Funding Statement.  “The funder provided support in the form of salaries for authors [insert relevant initials], but did not have any additional role in the study design, data collection and analysis, decision to publish, or preparation of the manuscript. The specific roles of these authors are articulated in the ‘author contributions’ section.”  If your commercial affiliation did play a role in your study, please state and explain this role within your updated Funding Statement.  b. Please also provide an updated Competing Interests Statement declaring this commercial affiliation along with any other relevant declarations relating to employment, consultancy, patents, products in development, or marketed products, etc.  Within your Competing Interests Statement, please confirm that this commercial affiliation does not alter your adherence to all PLOS ONE policies on sharing data and materials by including the following statement: "This does not alter our adherence to PLOS ONE policies on sharing data and materials.” (as detailed online in our guide for authors [http://journals.plos.org/plosone/s/competing-interests](about:blank)) . If this adherence statement is not accurate and there are restrictions on sharing of data and/or materials, please state these. Please note that we cannot proceed with consideration of your article until this information has been declared.  c. Please include both an updated Funding Statement and Competing Interests Statement in your cover letter. We will change the online submission form on your behalf.  Please know it is PLOS ONE policy for corresponding authors to declare, on behalf of all authors, all potential competing interests for the purposes of transparency. PLOS defines a competing interest as anything that interferes with, or could reasonably be perceived as interfering with, the full and objective presentation, peer review, editorial decision-making, or publication of research or non-research articles submitted to one of the journals. Competing interests can be financial or non-financial, professional, or personal. Competing interests can arise in relationship to an organization or another person. Please follow this link to our website for more details on competing interests: [http://journals.plos.org/plosone/s/competing-interests](about:blank) | Collaborative Care Systems Finland (CCSF) is a social innovation enterprise classified as a small and medium enterprise (SME) partner in the SMART2D Consortium. Similar to other partners in the project, its work was funded by the European Commission Horizon 2020 grant. Apart from this project, CCSF provides services for fee in the form of consultation and training for health care organisations and professionals in implementing behavior change science into practice in health promotion and disease prevention and self-management support.  The funding statement and the competing statements have been updated and also inserted in the cover letter:  Funding Statement:  This study is part of the SMART2D project supported by the EU Horizon 2020 Health Coordination Activities (Grant Agreement No 643692), under call HCO-05-2014 (Global Alliance for Chronic Diseases: Prevention and treatment of type 2 diabetes) URL: <https://ec.europa.eu/programmes/horizon2020/en/h2020-sections-projects>; and partially funded by Region Stockholm’s Public Healthcare Services Administration (Hälso- och sjukvårdsförvaltningen), URL: <https://www.sll.se/om-regionstockholm/Organisation/forvaltningar/halso-och-sjukvardsforvaltningen/>. The funders had no role in study design, data collection and analysis, decision to publish, or preparation of the manuscript. The funders provided support for part of the salaries of authors [LT, KSA, JÁA, PA, HMA and MD], but did not have any additional role in the study design, data collection and analysis, decision to publish, or preparation of the manuscript. The specific roles of these authors are specified in the ‘author contributions’ section.  Competing Interests Statement:  Collaborative Care Systems Finland (CCSF) is a social innovation enterprise classified as a small and medium enterprise (SME) partner in the SMART2D consortium. Similar to other partners in the project, its work was funded by the European Commission Horizon 2020 grant. Apart from this project, CCSF provides services for fee in the form of consultation and training for health care organisations and professionals in implementing behaviour change science into practice in health promotion and disease prevention and self-management support. CCSF as a commercial body had no role in the study design, data collection and analysis, decision to publish, or preparation of the manuscript; it does not alter our adherence to PLOS ONE policies on sharing data and materials. |
| 5.) Please include captions for your Supporting Information files at the end of your manuscript, and update any in-text citations to match accordingly. Please see our Supporting Information guidelines for more information: [http://journals.plos.org/plosone/s/supporting-information](about:blank) | Captions have been added according to Supporting Information guidelines.  Page 21, lines 583 - 587. |
| Abstract: it would be good to have participant numbers in the method, and a brief description of who the participants were: for example, age, gender and SES in the results. The conclusion could flow better. | Text added in the abstract: Ratings from 49 participants (19 diabetes and 30 at high risk) in ages 38-65 were analyzed.  Page 2, lines 50 - 51 |
| Introduction: it would be good to reference the new scale in the second aim. | Second aim was revised: 2) determine the acceptability of the intervention among participants living with diabetes or having a high risk of developing diabetes in socioeconomically disadvantaged areas in Stockholm using the newly developed tool based on the TFA.  Page 4, lines 115 - 118 |
| Method: How long is each intervention session? | Text added: The median duration per session was 17 minutes (range: 9-35 min) (Table 3). Table added on duration of each session: **Table 3. Duration per session** Session \| Median duration (50%) \| Range (25%-75%) \| Observations; 1 \| 11 \| 9 - 12 \| 45; 2 \| 19 \| 15 - 23 \| 47; 3 \| 22.5 \| 18 - 27 \| 44; 4 \| 19 \| 16 - 25 \| 33; 5 \| 20 \| 13.5 -25.5 \| 32; 6 \| 16.5 \| 12 - 23.5 \| 28; 7 \| 19.5 \| 15 - 23 \| 26; 8 \| 19 \| 13 - 27 \| 24; 9 \| 26 \| 21 - 35 \| 29 Page 7, lines 170 - 173 |
| Method: On lines 157 to 159, it is a bit confusing with regards to which language the sessions were presented in. | Method section revised: The sessions were delivered in Swedish, mostly using ‘easy Swedish’ with no use of medical or technical terms. When the participant’s Swedish language skills were too limited to receive the intervention in Swedish, the intervention was delivered in the participants’ native language by language skilled facilitators in Arabic, Somali or Spanish. Some participants preferred to have the intervention delivered in English, which could be offered by all facilitators.  Page 7, lines 175 -180. |
| Method: More detail is required about the face to face meetings (for example, how often/how long?). | Method section revised: In addition, two physical meetings per study site were arranged in the local community where the participants had the opportunity to meet each other, the facilitators, representatives from primary care, collaborating community organizations, experts or practitioners in diabetes, diet and physical activity, and other SMART2D team members. The meetings lasted approximately 3,5 hours each.  Pages 7-8, lines 192 - 196 |
| Method: In Table 2, Session 4, is the change over the years in relation to individual or population physical activity? | Table 2 session 4 clarified: Physical activity through the life course.  Page 7, lines 170 - 173 |
| Method: The data analysis only appears to describe factor analysis and not application of the tool to assess acceptability (or if it does do the latter then could this be stated more explicitly?) | Application of the tool is clarified: Likert summated scales were calculated for each participant within the identified construct from the EFA. Since the scales had a different number of items per each construct, the sum-scales were standardized to range from 0 to 100. [28]. Wilcoxon rank-sum test was conducted to detect differences between the sum-score for each domain and socio-demographic characteristics.  Page 9, lines 245 - 249. |
| Results: it would be good to have employment status and income level for participants presented. And, if appropriate, do some analysis of the factor score distribution in relation to these. | Employment status and income level for participants have been included in table 4. Page 10-11, lines 279-283  Since there were not significant differences, it was not appropriate to conduct further analysis on the factor score distribution in relation to these variables.  Text added: The unemployment rate was higher in the high-risk group which was also shown in the lower monthly income level in this group. No significant differences were found in employment status and income level between the participant groups. Therefore, no further analysis on the factor score distribution in relation to these variables were conducted. Page 10, lines 271 -275. |
| Discussion: line 289, I am not sure of the point of discussing smiley faces – could its relevance be made clearer?  It would have been useful to have some more detail of studies 44 and 45.  Given the low participant number, should the study be repeated with more participants to see if the results are replicated? | The sentence on smiley faces has been deleted.  Text expanded regarding refs 44 and 45 (original numbers) and one reference added:  Similar findings were found in other studies showing higher participation numbers for older compared to younger participants in lifestyle interventions for diabetes prevention [44]. Older participants are also more likely to adhere to laboratory tests and have higher self-monitoring rates and adherence for taking medication [44, 45]. Younger participants have also shown to have poorer glucose control and persons with early‐onset of T2D in younger ages have a higher prevalence of most clinical and behavioral risk factors, such as physical inactivity, tobacco smoking and obesity [46].  Page 17, lines 404 – 409.  We agree that the study should be repeated to see if the results are replicated. A sentence was added: The study would benefit from being repeated with more participants to see if the results are replicated.  Page 17, line 425 |
| Line 199: Please specify the type of correlation used to estimate the correlation matrix and justify the choice. | Spearman's rank correlation coefficients were calculated since the sample was small and tends to be more robust against outliers. We have revised the manuscript to clarify: “Spearman's rank correlation coefficients, a non-parametric correlation test that is less susceptible to outliers”, were constructed between different items from the questionnaire.  Page 9, lines 234-236 |
| Lines 203-204: The KMO test was used but the criteria used to determine appropriateness has not been specified. | The following text has been revised to add clarity and the KMO value. A reference was also added: The Kaiser-Meyer-Olkin (KMO) test was performed to confirm the appropriateness of data for EFA (KMO = 0.6230).  Reference: Hair, J. F., Black, W. C., Babin, B. J., Anderson, R. E., & Tatham, R. L. (2006). Multivariate Data Analysis. New Jersey: Pearson University Press.  Page 9, lines 240-241 |
| Line 208: It is not clear what m and k stand for. This has not been defined in the analysis section. | The authors agree this text was confusing. It has now been revised with the following text: “Since the scales had a different number of items per construct, the sum-scales were standardized to range from 0 to 100”  Page 9, line 246-247 |
| Lines 208-209: Please clarify what differences were being tested using the Wilcoxon rank sum test and its role in evaluating the psychometric properties of the scale. | The following text in quotations was added for clarification: Wilcoxon rank-sum test was conducted to detect differences “between the sum-score for each domain and socio-demographic characteristics”  Page 9 lines 247-249 |
| Please justify why were unidimensionality and validity (content, criterion, construct, convergent and divergent) not evaluated. | These concepts were evaluated based on the recommendations provided by Hamed Taherdoost. *Validity and Reliability of the Research Instrument; How to Test the Validation of a Questionnaire/Survey in a Research*. International Journal of Academic Research in Management (IJARM), 2016, 5. ffhal-02546799f. The authors have clarified (below) and revised the manuscript where appropriate to make it more clear. Specifically:   1. **Content validity** was assessed as clarified in the text by adding: “The content validity of the scale was evaluated by four researchers in the areas of public health, complex behavior change interventions, implementation science and medical anthropology and subsequently piloted with four participants.” Page 8, lines 208 -210. 2. **Criterion validity**: Unfortunately, a gold standard does not exist to assess acceptability, one of the main drivers behind the development of this tool. Therefore, it was not possible to measure the tool against a gold standard. However, we assessed the criterion concurrent validity (i.e. the extent that a measure simultaneously relates to another measure that it is supposed to relate) with the Spearman's rank correlation. An additional text was added: “Criterion validity was determined through the Spearman's rank correlation coefficients, a non-parametric correlation test which is less susceptible to outliers, constructed between different items from the questionnaire. Page 9, lines 233 - 136. 3. **Construct validity** (convergent and divergent) was assessed using exploratory factor analysis. We have clarified this in the methods section by adding: “Exploratory factor analysis (EFA) was performed to assess the construct validity (discriminant and convergent validity) of the acceptability domains from the Sekhon framework” Page 9, lines 236 - 237.   Finally, the EFA is not an appropriate technique to test unidimensionality. However, it is a viable approach for deriving hypotheses regarding the possible number of factors underlying the item intercorrelations. Since there were three latent factors, this gives evidence to the multidimensionality of the data. However, the authors acknowledge the limitation of the data. Confirmatory factor analysis would be recommended on a larger, different data set. |
