# Supplementary material for: Application of the Theoretical Framework of Acceptability to assess a telephone-facilitated health coaching intervention for the prevention and management of type 2 diabetes
Source: PLoS One. 2022 Oct 6;17(10):e0275576. doi: 10.1371/journal.pone.0275576 (PMC9536591; doi:10.1371/journal.pone.0275576)
Supplement: Attachment — Submitted filename: Response to Reviewers_PLOSOne_Timm L. et al.docx [file pone.0275576.s007.docx]

Response to Reviewers

Thank you for your valuable comments. Please see our response and revisions below.

| **Comment from reviewer** | **Author response** | **Revision** |
| --- | --- | --- |
| Minor revision: Cite the statistical software used for the analysis. | Good point. We have now added the missing information. | Text added on page 9, lines 229-230  The statistical software package STATA version 15.1 was used to conduct the analysis. |

In addition, some changes have been made in the manuscript:

**Page 1**: The affiliation of the corresponding author has changed. Therefore, on the front page one department is added and the numbering is revised.

**Page 11 and 14**: Minor clarifications (formatting and added text, but no changes of numbers) have been done in Table 4 and Table 6.

**Page 16,** lines 113-315: In the discussion section, the sentence “The difference in age groups reported in table 6 showed that scores for this construct was significantly higher among younger persons compared with the older participants” is added.

**Page 17,** lines 346-349: The following clarification is made:

| Previous text | Current text after revision |
| --- | --- |
| The intervention was also perceived to be more burdensome among younger participants for reasons that are likely to be very similar. Similar findings were found in other studies showing higher participation numbers for older compared to younger participants in lifestyle interventions for diabetes prevention [44]. | Although the affective attitude and effectiveness was high among younger participants (Table 6), the intervention was perceived to be more burdensome among younger participants. Similar findings confirming a high burden for younger persons, were found in other studies showing higher participation numbers for older compared to younger participants in lifestyle interventions for diabetes prevention [44]. |
