# Supplementary material for: Application of the Theoretical Framework of Acceptability to assess a telephone-facilitated health coaching intervention for the prevention and management of type 2 diabetes
Source: PLoS One. 2022 Oct 6;17(10):e0275576. doi: 10.1371/journal.pone.0275576 (PMC9536591; doi:10.1371/journal.pone.0275576)
Supplement: Attachment — Submitted filename: Response to Reviewers.docx [file pone.0275576.s008.docx]

**Response to Reviewers**

Thank you for your valuable comments. Please see our response and revisions below.

Reviewer #3: 

Two suggestive amendments are as follows:
1. Exploratory Factor Analysis: Are you assuming that the five theoretical constructs are independent? Are the five constructs correlated with each other?

Response to suggestive amendment 1:

The items in the different theoretical constructs are correlated with each other as reported in the correlation matrix of the data. The original constructs are all part of the Theoretical Framework of Acceptability as discussed in the text. Further, the EFA has retained the most relevant items to our acceptability dimensions representing what we believe are the participants’ thinking and experience of the intervention.

2. Limitation of EFA: Originally, there were seven constructs developed for TFA. However, only five constructs appear to show the relevance to TFA. The EFA procedure is sensitive to the variations in the samples selected. In other words, how can investigators handle the potential biases introduced by the sample?

Response to suggestive amendment 2:

It is possible that with a larger sample we would have been able to keep a larger number of items and therefore a more detailed representation of the original seven constructs of the TFA. However, the sampling adequacy test showed that the data used in our analyses was adequate to perform EFA, the items included in this study and the obtained constructs were relevant and showed validity to our acceptability study.

Reviewer #5:

Methods: Please note the training of the trained facilitators specifically. Table 2, session content notes “peer” in the description. Did the study use peer coaches? If peers, it need to specifically state throughout that the intervention of interest is peer coaching. Peer coaching is not currently covered within the standardized definition of Health and Wellness Coaching, which is currently driving practice changes, including reimbursement, in the US Setting. (Wolever 2013 in Global advances in Health and Medicine; Wolever 2016 in BMC Health Services Research).

Response: Thank you for this valuable comment. It was not correct to use “peer” in Table 2. We have clarified this by replacing the word peer to the more adequate word care companion, which is also used in the descriptive text.

Please describe the amount of missingness led to surveys being excluded from the analysis (n=6).

Response: Revision on page 9. To clarify the amount of missingness, the number is corrected to 5 in the text, now corresponding to the numbers in the figure.

Results: I don’t think that “excluded from the acceptability study” is the right terminology. If I am reading methods correctly, only 6 were excluded due to missingness. The others chose not to participate in the acceptability study portion. Therefore, I think something like “intervention completed; declined acceptability study” is more appropriate. The 6 that didn’t fully complete the data collection of the acceptability study could still be labelled as excluded.

Response: Thank you for this comment, the terminology is revised both in the text and in the table for clarification (pages 10-11). Please note the comment above on the revised number.

We have considered the comments about introduction and discussion from Reviewer 5. These revisions are according to editors optional and we have chosen to leave these parts as they have been thoroughly revised earlier according to previous reviewers comments.

Line 71: Citation marks have been added to the first sentence.

Line 369: An ‘i’ was missing after the word interlinked and this is corrected.

Table 4 on page 1. ‘of’ has been changed to ‘or’.

_________________________________________________________________________
